# An Encapsulated Vitamin A Palmitate Powder With Improved Stability for Use in Food Fortification

**DOI:** 10.1002/fsn3.71237

**Published:** 2025-11-21

**Authors:** Samantha Brady, Elsa Abou Jaoude, Julie Wyns, Haisong Yang, Justyna Ebbesen, Elise Ivarsen, Julie Straub, Jérôme Vallejo, Don Chickering

**Affiliations:** ^1^ Particles for Humanity, PBC Cambridge Massachusetts USA; ^2^ LIS France Cérences France; ^3^ Eurofins Vitamin Testing A/S Vejen Denmark; ^4^ Straub Pharma Consulting, Inc. Winchester Massachusetts USA

**Keywords:** cooking, food fortification, methacrylate, stability, vitamin A, vitamin A deficiency

## Abstract

Vitamin A deficiency (VAD) is a serious health concern, particularly in low‐ and middle‐income countries. Food fortification is hindered by the instability of vitamin A. The objective was to develop a dry form of vitamin A palmitate (VAP) with improved stability compared to marketed products. This powder could be employed for large‐scale food fortification to help prevent VAD. VAP is emulsified with a variety of food‐grade ingredients, including basic methacrylate copolymer (BMC). A spray‐dried BMC‐encapsulated VAP (PFH‐VAP) was produced using laboratory‐scale equipment. Subsets were assessed for determination of the VAP content and VAP recovery after cooking in water. Another subset was subjected to accelerated stability testing. Two high‐performance formulations were produced at the pilot scale. The two most promising PFH‐VAP formulations and a commercially available vitamin A product were each incorporated into bouillon cubes, stored (40°C, 75% relative humidity), and subjected to stability testing. The optimized laboratory‐scale formulations exhibited > 90% VAP recovery after cooking. The pilot‐scale batches showed 95% and 65% VAP recoveries after cooking and accelerated stability testing, respectively. After 12 months of stability testing, 70% and 68% VAP recoveries were achieved for the pilot‐scale batches of PFH‐VAP‐fortified bouillon. A 15% VAP recovery was obtained from the bouillon fortified with a commercial product. PFH‐VAP demonstrated a substantial stability advantage over an existing commercial formulation of VAP in a proof‐of‐concept food vehicle (bouillon). The use of PFH‐VAP could increase the nutritional and health benefits of vitamin A‐fortified foods and condiments.

## Graphical abstract

Food fortification is hindered by the instability of vitamin A. Encapsulating vitamin A palmitate (VAP) in a basic methacrylate copolymer (BMC) and spray drying to form PFH‐VAP resulted in a substantial stability advantage over comparator material when incorporated into bouillon and stored at 40°C/75% RH. After 12 months, PFH‐VAP retained 70% of vitamin A versus 15% for the comparator, supporting its potential to increase the nutritional benefits of fortified foods.

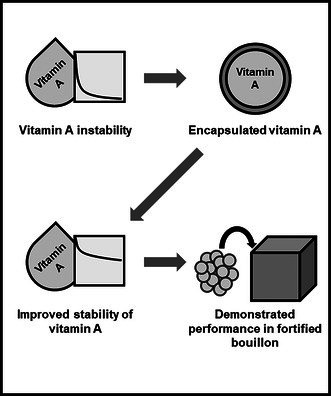

## 1 Introduction

Vitamin A deficiency (VAD) is associated with numerous health problems, including adverse effects on child growth and development (Akhtar et al. 2013; Ssentongo et al. 2020), childhood blindness (Akhtar et al. 2013; Bailey et al. 2015), immune system disorders (Bono et al. 2016; Pino‐Lagos et al. 2010; Ross 2012), and a greater susceptibility to infection (Bailey et al. 2015). Increased rates of VAD have been reported in low‐ and middle‐income countries (Stevens et al. 2022; World Health Organization 2009; Zhao et al. 2022), particularly in children under 5 years of age (Zhao et al. 2022).

Although fortifying commonly consumed foods with vitamin A is one potential means to address VAD (Bailey et al. 2015; Iwuozor et al. 2022; World Health Organization 2006), vitamin A degrades when exposed to heat, light, or oxygen (Gonçalves et al. 2016; Hemery et al. 2018; Maurya et al. 2022; Ohanenye et al. 2021; Uchendu and Atinmo 2016). This instability has impeded food fortification efforts (Chepkoech et al. 2022; Pinkaew et al. 2012), and is especially problematic in high‐temperature regions such as sub‐Saharan Africa, where VAD rates are high (Hemery et al. 2018; Ohanenye et al. 2021; Zhao et al. 2022). Furthermore, the levels of vitamin A in fortified foods often decline during their time within the supply chain, leading to reduced shelf lives. Increased costs are incurred when extra vitamin A is added to account for this loss.

Dry vitamin A palmitate (VAP) products that contain 250,000 international units per gram of this compound (i.e., VAP250 products) are commonly used to fortify dry foods with vitamin A. Currently, they are available in various formulations including encapsulated products (Maurya et al. 2022). Previous research has shown that the microencapsulation of vitamin A has a beneficial effect on its stability (Anselmo et al. 2019; Gonçalves et al. 2016; Maurya et al. 2022; Ohanenye et al. 2021; Tang et al. 2022; Xu and Watson 2021). Basic methacrylate copolymer (BMC) has been used for many years as a pharmaceutical tablet coating, and is considered safe for use in micronutrient encapsulation for food fortification (World Health Organization 2018). Importantly, nonclinical studies and a bioavailability study in healthy volunteers demonstrated that the BMC‐encapsulation of vitamin A did not negatively impact its absorption (Anselmo et al. 2019; Tang et al. 2022). These studies demonstrated small‐scale feasibility of VAP protection with BMC, but the formulations used in these studies were not made with materials included in the General Standard for Food Additives (GSFA) list (Codex Alimentarious International Food Standards 2023) at manufacturing scales large enough to support commercialization.

With these considerations in mind, this report describes the development of PFH‐VAP, a BMC‐encapsulated VAP composition that is intended to improve the effectiveness of food fortification programs using VAP250 products. Optimization of the composition and preparation process is employed to produce a food‐grade VAP formulation with enhanced stability. Proof‐of‐concept is subsequently demonstrated through storage stability evaluations of fortified bouillons.

## 2 Methods

### 2.1 Overview

The process and formulation were developed using laboratory‐scale equipment (batch size: 10–200 g). Formulations were optimized to maximize stability, for cost, and to include only food‐grade ingredients included in the GSFA. The production process was refined to ensure suitability for large‐scale equipment.

Over 130 batches were spray‐dried and analyzed. Formulations were prioritized for testing in the bouillon based on their evaluated performances, and to provide a breadth of formulation options during the initial optimization phase. The formulations were subsequently prioritized based on their performances in the bouillon samples over time. Bouillon was selected as a proof‐of‐concept food vehicle because it is widely consumed in many areas, including West Africa, where VAD is common. Additional details regarding the equipment and materials employed are provided in Tables S1 and S2, respectively.

### 2.2 Preparation of Laboratory‐Scale Batches During Optimization

The PFH‐VAP formulation was initially optimized on the laboratory scale. The formulation was iterated by changing the variables and measuring the resulting performance over time, as summarized in Table 1. Vitamin A materials commonly contain antioxidants that preserve the vitamin's stability. Thus, for the purpose of this study, butylated hydroxyanisole (BHA), butylated hydroxytoluene (BHT), and tocopherol (TOC) were incorporated into the tested formulations. Notably, TOC is a naturally occurring alternative to BHA and BHT, which are synthetic compounds. BHA, BHT, and TOC are commonly used for stabilizing VAP; other natural antioxidants were not considered.

**TABLE 1:** Iterated variables during laboratory‐scale development and optimization.

Variable	Iterations
Formulation ingredients	Type and combination of acid Presence of surfactant Type and combination of antioxidants Type of modified starch
Ingredient ratios	Amount of acid added Amount of antioxidant (s) added Ratio of modified starch to maltodextrin Ratio of VAP to BMC
Liquid preparation	pH of BMC dispersion Order of ingredient addition Material in which antioxidant (s) were added (e.g., in the VAP oil, preemulsion, and/or final emulsion) Method of acid addition (e.g., titration or bulk addition) Agitation conditions (e.g., volume, mixing speed, temperature, mixing time) Homogenization conditions (e.g., rotor stator or high‐pressure homogenizer) Dry matter of the liquid preparations, including the final emulsion for spray drying
Drying process	Spray dryer Spray dryer with fluidized bed of native starch Type and amount of material used to coat particles on the fluid bed dryer Temperature, flow rate, and pressure conditions on the spray dryer

Abbreviations: BMC, basic methacrylate copolymer; VAP, vitamin A palmitate.

### 2.3 Laboratory‐Scale Preparation of PFH‐VAP Based on the Optimized Formulations

Optimized laboratory‐scale formulations of PFH‐VAP were prepared by spray drying of oil‐in‐water emulsions. More specifically, an initial solution was prepared by adding L‐ascorbic acid (20.5 g) to ultrapure water (~400 g), resulting in a solution of pH < 5.5. Subsequently, BMC (104.0 g) was added, and the resulting dispersion was agitated first for 3 h at 35°C and then for ~18 h at 20°C.

After this time, VAP oil (104.0 g; prestabilized with BHT or TOC) was preheated to 50°C to promote liquification. For batch A, BHA (10.4 g) was added to the BHT‐stabilized VAP, while for batch B, TOC‐stabilized VAP was used and no additional BHA was added. In addition, maltodextrin DE19 (14.8 g), HI CAP 100modified starch (133.3 g), and L‐ascorbic acid (20.5 g) were combined in ultrapure water (~200 g), mixed for 30 min under magnetic stirring, and heated to 50°C. The above‐prepared VAP oil was added to the resulting mixture, agitated with a magnetic stirrer bar, and emulsified using an APV Model 2000 (SPX Flow Inc.) high‐pressure homogenizer for ~13 min at 500/50 bar (inlet pressure/outlet pressure). The BMC dispersion was then added to the vessel and stirred using an Ultra‐Turrax T25 high‐shear mixer (IKA Works Inc.) at 15,000 rpm for 5 min.

The resulting ~1 kg oil‐in‐water emulsion was dried on a Minilab RC fluidized bed dryer charged with native starch powder (100.0 g). Drying was performed using an inlet air temperature of 75°C for 43 min, a liquid feed rate of 690 g/h, a liquid feed peristaltic pump speed of 45 rpm, an atomizing air pressure of 1 bar, and an inlet air volume flow rate of 65 m^3^/h.

### 2.4 Preparation of Pilot‐Scale Batches of the PFH‐VAP Microparticles

Pilot‐scale batches of PFH‐VAP (powder batch size ≈80 kg) were prepared by spray drying the 250–300 kg oil‐in‐water emulsions, as outlined in Table 2.

**TABLE 2:** PFH‐VAP pilot‐scale batch preparation.

Step	Batch C	Batch D
**VAP preparation**	VAP oil (17.5 kg) (unstabilized) was heated in a 70°C‐heating belt for ≥ 12 h	VAP oil (11.3 kg) purchased prestabilized with TOC was heated in a 70°C‐heating belt for ≥ 12 h
	BHA (1 kg) and BHT (1 kg) were added	—
**Preparation of the BMC dispersion**	L‐Ascorbic acid (3.5 kg) was added to reverse osmosis water (60.0 kg), resulting in a solution with pH < 5.5	L‐Ascorbic acid (2.3 kg) was added to reverse osmosis water (45.0 kg), resulting in a solution with pH < 5.5
	BMC (17.5 kg) was added, and the resulting dispersion was agitated for 5 h at room temperature	BMC (11.3 kg) was added, and the resulting dispersion was agitated for ~3 h at room temperature
**Stabilizer preparation**		
Amount of reverse osmosis water	~143.0 kg	~130.0 kg
Stabilizers added to water	Maltodextrin DE19 (33.7 kg)CAPSUL TA‐modified starch (22.5 kg)L‐Ascorbic acid (3.5 kg)	Maltodextrin DE19 (33.4 kg)CAPSUL TA‐modified starch (14.5 kg)L‐Ascorbic acid (2.2 kg)
Mixing method	Tank agitator at 800 rpm and recirculation in the tank	
Mixing time and heat	~40 min Heated to 50°C	~60 min Heated to 50°C
**VAP addition to the stabilized mixture**		
Amount of VAP	19.5 kg of the VAP oil/antioxidant mixture	11.3 kg of the VAP oil
Mixing and emulsification	Tank agitator at 800 rpm and recirculation in the tank, then MG2‐350S HPH recirculated in a loop at the flow rate of the HPH (estimated 340 kg/h) for ~2.5 h at 340/150 bars	
**BMC dispersion addition and mixing**	The BMC dispersion was added and stirred with a tank agitator (700 rpm; no recirculation in the tank) for ~10–30 min	The BMC dispersion was added and stirred with a tank agitator (700 rpm; no recirculation in the tank) for 50 min
**Emulsion drying**		
Equipment	Entropie Serit SME 180 AB1 pilot multistage spray dryer with an internal fluid bed	
Time	~10 h	~8 h
Inlet/outlet air temp of the spray dry tower	140°C–150°C/64°C–66°C	150°C–160°C/68°C
Inlet air temp of the internal fluid bed dryer	60°C	60°C
Recirculation	Fines were recirculated to the internal fluid bed	
**Final step**	The powder was passed through a 2 mm sieve prior to packaging	

Abbreviations: BHA, butylated hydroxyanisole; BHT, butylated hydroxytoluene; BMC, basic methacrylate copolymer; HPH, high‐pressure homogenizer; TOC, tocopherol; VAP, vitamin A palmitate.

### 2.5 Determination of the Vitamin A Loading for the Microparticles

The VAP powder (20 mg, VAP250) was added to water (0.2 mL) and vortexed for 1 min. Subsequently, tetrahydrofuran (THF) containing 0.1% BHT (1.8 mL) was added, and the mixture was vortexed for2 min prior to centrifugation at 8000 rcf for 5 min. Reversed‐phase high‐performance liquid chromatography (RP‐HPLC) was performed using an aliquot (0.2 mL) of the supernatant diluted 10× in acetonitrile (ACN) containing 0.1% BHT. After vortexing for 2 min, the sample was filtered through a 0.22 μm polyvinylidene fluoride (PVDF) filter. All samples were prepared in triplicate.

### 2.6 Reversed‐Phase HPLC Quantification of Vitamin A Palmitate

VAP was quantified by means of RP‐HPLC (UltiMate 3000, Thermo Fisher Scientific) using methanol/water (97:3) as the mobile phase at a flow rate of 1.5 mL/min. The samples were passed through a C18 reversed‐phase column (Supelco C18 Discovery, 150 × 4.6 mm, 5 μm) using a 20–100 μL injection volume and were analyzed at 325 nm using an ultraviolet–visible detector. Two peaks were observed for VAP, namely a major symmetric peak at a mean (± standard deviation) retention time of 27.1 (± 2.2) min for *trans*‐retinyl palmitate, and a minor peak with a mean (± standard deviation) retention time of 25.4 (± 2.1) min for *cis* retinyl palmitate. Standard stock solutions containing 0.1% BHT and 4.5–5.3 mg/mL VAP were prepared in ethanol. To determine the VAP content, the sample peak was integrated and calculated against a linear calibration curve prepared using a minimum of six calibration standards (concentration range of 1.0–500 μg/mL).

### 2.7 Preparation of Vitamin A‐Fortified Bouillon Cubes for the Laboratory‐Scale Study

Bouillon cubes fortified with nominal amounts of iron (6 mg/g) and iodine (0.03 mg/g) (Maggi, Nestle, Nigeria) were unwrapped and ground into a coarse powder using a food processor (Cuisinart PowerPrep Plus, 14 Qt) equipped with an S‐shaped stainless steel chopping blade. The VAP‐containing microparticle powders were weighed and added to the bouillon powder at a concentration of 673 μg/g bouillon, which represents 154% of the recommended daily vitamin A intake level (Lewis 2019). The daily bouillon intake was assumed to be consistent with one serving of bouillon (3.33 g) listed on Nestle Maggi Star bouillon. The fortified bouillon powders were mixed until uniformly dispersed. The cubes were then reformed using a tablet press (Model C benchtop manual press, Carver Inc.) and a 15 mm cube‐shaped punch and die set composed of 7S ESR tool steel (Natoli Engineering). For each composition, a sample (4 g) of the fortified bouillon powder was added to the die cavity, the upper punch was placed on the die, and pressure (0.5 ton) was applied for 10 s using the tablet press. An ejector die and a manual arbor press were used to remove the cubes, which were stored refrigerated in vacuum‐sealed aluminum packaging until further use.

### 2.8 Preparation of Vitamin A‐Fortified Bouillon Cubes for the Pilot‐Scale Study

Bulk Maggi bouillon powder fortified with nominal amounts of iron (0.6 mg/g) and iodine (0.03 mg/g) was mixed with the VAP‐containing powders at a concentration of 350 μg VAP per gram of bouillon, which represents 80% of the recommended daily vitamin A intake level (Lewis 2019). The daily bouillon intake was assumed to be consistent with one serving of bouillon (3.33 g) listed on Nestlé Maggi Star bouillon. The vitamin A concentration was decreased relative to the laboratory‐scale study so that it was below the daily total vitamin A intake level. A sample subset was also produced by adding further iron to the bouillon powder at a concentration of 2.6 mg/g. The fortified bouillon powder was blended manually by hand followed by blending in a Kmix 750 stand mixer (Kenwood) until a uniform mixture was obtained. Cubes (11 g) were formed using a P40 rotary press (Bonals Technologies) and wrapped with an oriented polypropylene film using a BCW3 wrapping machine (Theegarten‐Pactec GmbH & Co. KG). The cubes were stored refrigerated in polypropylene food‐storage containers until required for further use.

### 2.9 Determination of the Vitamin A Palmitate Loading in Bouillon

The bouillon cubes were reconstituted to a concentration of 91 mg/mL in water. A 4 mL sample was transferred to a 50 mL polypropylene centrifuge tube, frozen at −80°C, and lyophilized for ≥ 24 h. After the addition of water (0.3 mL), the mixture was vortexed for 1 min, and an aliquot of 0.1% BHT in THF (2.7 mL) was added. The resulting mixture was vortexed once again for 3 min and subjected to centrifugation at 8000 rcf for 5 min. After diluting an aliquot (0.5 mL) of the supernatant by 10× in ACN containing 0.1% BHT, the mixture was vortexed for 1 min, filtered through a 0.22 μm PVDF filter, and analyzed by RP‐HPLC.

### 2.10 Stability Studies

#### 2.10.1 Stability Test for the Microparticles in Heated Water (Cooking)

The VAP‐containing powder (20 mg) was added to water (2.0 mL) and vortexed for 2 min. The suspension was then heated to 90°C and maintained at this temperature (±1°C) for 2 h while mixing at 500 rpm using an Eppendorf ThermoMixer C. The mixture was then cooled to room temperature (20°C ± 2°C), frozen at −80°C, and lyophilized for ≥ 24 h. The vitamin A content of the resulting powder was analyzed using the methods described above to determine the vitamin A loading for the microparticles. The results were reported as a percentage of the amount measured prior to cooking.

#### 2.10.2 Accelerated Stability Test for the Microparticles

A sample of the VAP‐containing powder (1.0 g) was evenly distributed in a 4.5 cm‐diameter open Petri dish to maximize oxygen exposure. The samples were stored at 40°C at an ambient relative humidity (RH) near 20% in a Memmert UF75 universal oven for ~28 d. Although the samples were stored in a dark oven, they were not protected from ambient light when the oven door was opened for sampling at predetermined time points. Thus, they were handled quickly to minimize light exposure. Sampling was performed on the first, second, third, fourth, and/or fifth week to determine the vitamin A content. The results were reported as a percentage of the amount measured before storage.

#### 2.10.3 Stability Testing of the Bouillon Cubes

Bouillon cubes were placed in individual, open 50 mL polypropylene centrifuge tubes and stored at 40°C and 75% RH in accordance with the International Council for Harmonization (ICH) guidelines for accelerated stability testing for all world zones (International Conference on Harmonization 2003). The VAP content and stability were measured at predetermined time points (i.e., 0, 1, 2, 3, 6, 9, 12, 18, and 24 months). The results obtained at each time point were compared with the VAP concentration measured at the start of the study (time = 0). Triplicate cube samples were assessed for each study group at the various time points.

#### 2.10.4 Stability Testing of Bouillon in Heated Water (Bouillon Cooking)

Bouillon cubes were reconstituted to a concentration of 91 mg/mL in water. A 250 mL beaker containing water (150 mL) and a magnetic stirrer bar was heated to 90°C and maintained at this temperature (±1°C). A sample of the reconstituted bouillon (40 mL for laboratory scale; 50 mL for pilot scale) was transferred to the beaker, stirred for 2 h at 250 rpm, and loosely covered with aluminum foil. The temperature of the suspension was measured every 30 min. To account for evaporation of the water, the material in the beaker was weighed prior to sampling and water was added as required. After 2 h, a sample of the broth (10 mL) was transferred to a 50 mL polypropylene tube, frozen at −80°C, and lyophilized for ≥ 24 h. After this time, water (0.3 mL) was added and the mixture was vortexed for 1 min. Subsequently, 0.1% BHT in THF (2.7 mL) was added, and the mixture was vortexed for 3 min prior to centrifugation at 8000 rcf for 5 min.

After diluting an aliquot (0.5 mL) of the supernatant by 10× in ACN containing 0.1% BHT, the mixture was vortexed for 1 min, filtered through a 0.22 μm PVDF filter, and analyzed by RP‐HPLC.

### 2.11 Data Analysis

The VAP content measured by RP‐HPLC was further analyzed in Microsoft Excel. Three replicates were used for each measurement unless otherwise stated. Values are reported as mean ± standard deviation. Graphs were generated using the GraphPad Prism software.

### 2.12 Particle Characterization

To assess microparticle morphology, specimens were coated with gold and palladium, and scanning electron microscopy (SEM) was performed at 1000× magnification (Phenom Pure+, Thermo Fisher Scientific). The microparticle size was determined using a Mastersizer 3000 (Malvern PANalytical) instrument. To determine the size distribution of the dry microparticles, an MS3000 dry sample dispersion unit was employed.

## 3 Results

**FIGURE 1:**
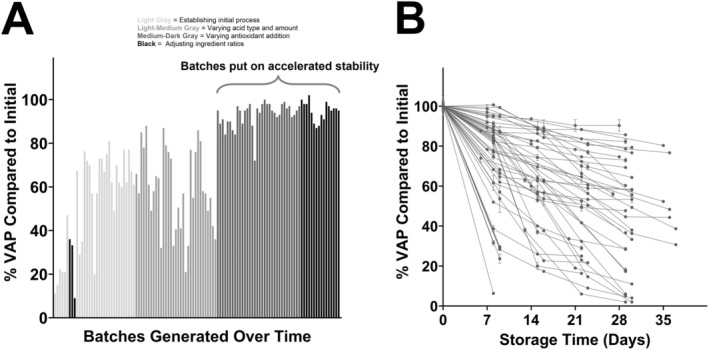
PFH‐VAP optimization at the laboratory scale. (A) percentage of VAP remaining after cooking the PFH‐VAP microparticles relative to the initial amount. Bars are color coded according to formulation compositional change as follows: Light gray bars represent batches produced to establish the initial process, light‐medium gray bars represent batches produced with varying acid types and amounts, dark‐medium gray bars represent batches produced with varying antioxidant addition, and black bars represent batches produced with adjustments to ingredient ratios. Later batches with higher recoveries were subjected to accelerated stability testing (40°C and ambient relative humidity). (B) Accelerated stability results for each batch of PFH‐VAP microparticles over time. Initially, the optimized batches demonstrating good cooking recoveries did not always yield good accelerated stability performances. This metric was improved through optimization. VAP, vitamin A palmitate.

More than 130 batches were manufactured at the laboratory scale, and a subset was subjected to accelerated stability testing (Figure 1). The cooking performance was found to improve during development as the formulations were iterated, with batches produced later in development recovering more vitamin A relative to batches made earlier in development. High‐performance formulations were differentiated further during the accelerated stability testing. These results indicate that the critical factors included tuning the type and amount of acid used to disperse the BMC prior to emulsification and the addition of antioxidants. Two final formulations were selected for continued development at the pilot scale (denoted PFH‐VAP250‐101 and PFH‐VAP250‐102); all ingredients were compliant with the Codex General Standard for Food Additives (GFSA) (Codex Alimentarious International Food Standards 2023). PFH‐VAP250‐101 contained both BHA and BHT antioxidants and exhibited the best performance using the above stability tests. PFH‐VAP250‐102 contained the antioxidant TOC and performed acceptably, but not as well as PFH‐VAP250‐101. However, a decision was made to continue with both formulations because downstream stakeholders may prefer a naturally occurring antioxidant such as TOC. Characteristics of the PFH‐VAP microparticles from representative lab‐scale batches made with these two formulations are summarized in Table 3. The loading efficiency was higher than 100% because the theoretical loading was calculated assuming that all the fluidized starch used during drying was incorporated into particles, but there was likely loss of starch to the equipment and exhaust.

**TABLE 3:** PFH‐VAP loading, size, and span.

Batch number	Batch information	Theoretical loading (% w/w)	Mesaured loading (% w/w)	Loading efficiency (%)	Particle D50 (μm)	Span
PFH‐VAP250‐101	Laboratory scale	15.3	15.7	102.6	502	1.2
	Pilot scale	17.5	16.1	92.0	102	1.6
PFH‐VAP250‐102	Laboratory scale	15.7	16.5	105.1	395	1.2
	Pilot scale	15.1	13.5	89.4	86	1.5

**FIGURE 2:**
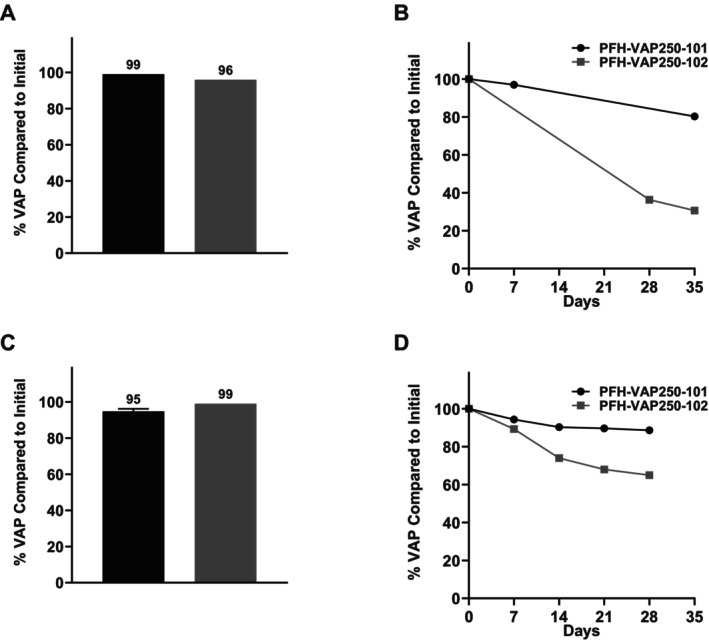
Performances of selected formulations after cooking and under accelerated stability conditions on the laboratory and pilot scales. (A) Laboratory‐scale performance: Percentage of VAP remaining compared to the initial amount after cooking the laboratory‐scale PFH‐VAP microparticles. (B) Laboratory‐scale performance under accelerated stability conditions (40°C and ambient relative humidity). (C) Pilot‐scale performance: Percentage of VAP remaining compared to the initial amount after cooking the pilot scale PFH‐VAP microparticles. (D) Pilot‐scale performance under accelerated stability conditions (40°C and ambient relative humidity). Black bars represent PFH‐VAP250‐101 and gray bars represent PFH‐VAP250‐102. The error bars represent the standard deviation from the mean. For both formulations, the laboratory‐ and pilot‐scale products showed similar performances after cooking. In addition, both formulations showed good accelerated stabilities on the laboratory scale and superior stabilities at the pilot scale.

Both pilot batches of the PFH‐VAP microparticles demonstrated good stabilities after cooking in water and under the accelerated conditions designed to induce oxidative stress. More specifically, the pilot formulations exhibited recoveries of 95% and 65% after the cooking and accelerated stability tests, respectively (Figure 2). These results match or are higher than the corresponding laboratory‐scale results. In addition, it was found that PFH‐VAP250‐102 exhibited a superior accelerated stability performance when fabricated at the pilot scale. This could be due to improved emulsion quality on pilot‐scale equipment, which may result in better encapsulation of vitamin A. Characteristics of the PFH‐VAP microparticles from representative batches are summarized in Table 3. The loading efficiency was between 89% and 92% and was lower than the corresponding lab‐scale batches. This is likely because no fluidized starch was added for pilot‐scale drying and some loss is expected through VAP degradation during processing.

**FIGURE 3:**
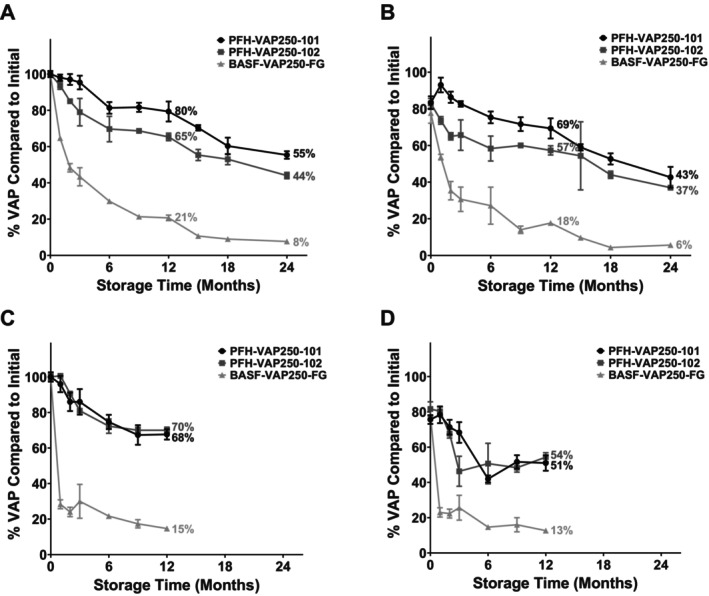
Results in bouillon after storage under harsh conditions (40°C/75% relative humidity) and after storage and cooking (90°C water for 2 h). (A) Percentage of VAP remaining relative to the initial amount after storing the laboratory‐scale PFH‐VAP microparticles and commercial products. (B) Percentage of VAP remaining compared to the initial amount after storing and cooking the laboratory‐scale PFH‐VAP microparticles and commercial products. (C) Percentage of VAP remaining compared to the initial amount after storing the pilot‐scale PFH‐VAP microparticles and products. (D) Percentage of VAP remaining compared to the initial amount after storing and cooking the pilot‐scale PFH‐VAP microparticles and commercial products. The error bars represent the standard deviation from the mean. Both the laboratory‐ and pilot‐scale PFH‐VAP formulations demonstrated improved stabilities compared to the commercial formulations when stored in bouillons.

**FIGURE 4:**
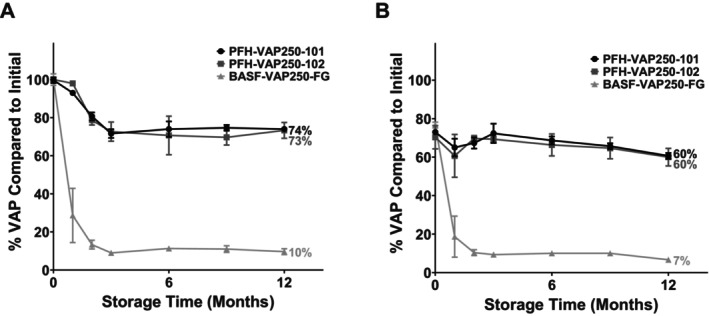
Results in bouillon with added iron after storage under harsh conditions (40°C/75% relative humidity) and after storage and cooking (90°C water for 2 h). (A) Percentage of VAP remaining relative to the initial amount after storing the pilot‐scale PFH‐VAP microparticles and commercial products. (B) Percentage of VAP remaining compared to the initial amount after storing and cooking the pilot‐scale PFH‐VAP microparticles and commercial products. The error bars represent the standard deviation from the mean. Both PFH‐VAP formulations demonstrated improved stabilities compared to the commercial formulations when stored in bouillon containing ferrous pyrophosphate.

Bouillon made with the laboratory‐scale and pilot‐scale formulations was subsequently tested in a series of stability studies. As presented in Figure 3, both the laboratory‐scale and pilot‐scale PFH‐VAP samples exhibited superior stabilities in the bouillons than a commercial VAP250 product after storage under harsh conditions (40°C/75% RH) and after storage and cooking. After 24 months of storage, 55% and 44% of the VAP were recovered from the laboratory‐scale PFH‐VAP batches, as opposed to 8% for the commercial product (unpaired *t*‐test, *p* < 0.05). After 24 months of storage followed by cooking, 43% and 37% of the VAP were recovered from the laboratory‐scale PFH‐VAP batches, as opposed to 6% for the commercial product (unpaired t‐test, *p* < 0.05). After 12 months of storage, 70% and 68% of the VAP were recovered from the pilot‐scale PFH‐VAP batches, as opposed to 15% for the commercial product (unpaired *t*‐test, *p* < 0.05). After 12 months of storage followed by cooking, 54% and 51% of the VAP were recovered from the pilot‐scale PFH‐VAP batches, as opposed to 13% for the commercial product (unpaired *t*‐test, *p* < 0.05). Following the additional iron fortification of the bouillon cubes, the stability of PFH‐VAP exceeded that of the commercial VAP product after storage under harsh conditions and after storage followed by cooking (Figure 4).

**FIGURE 5:**
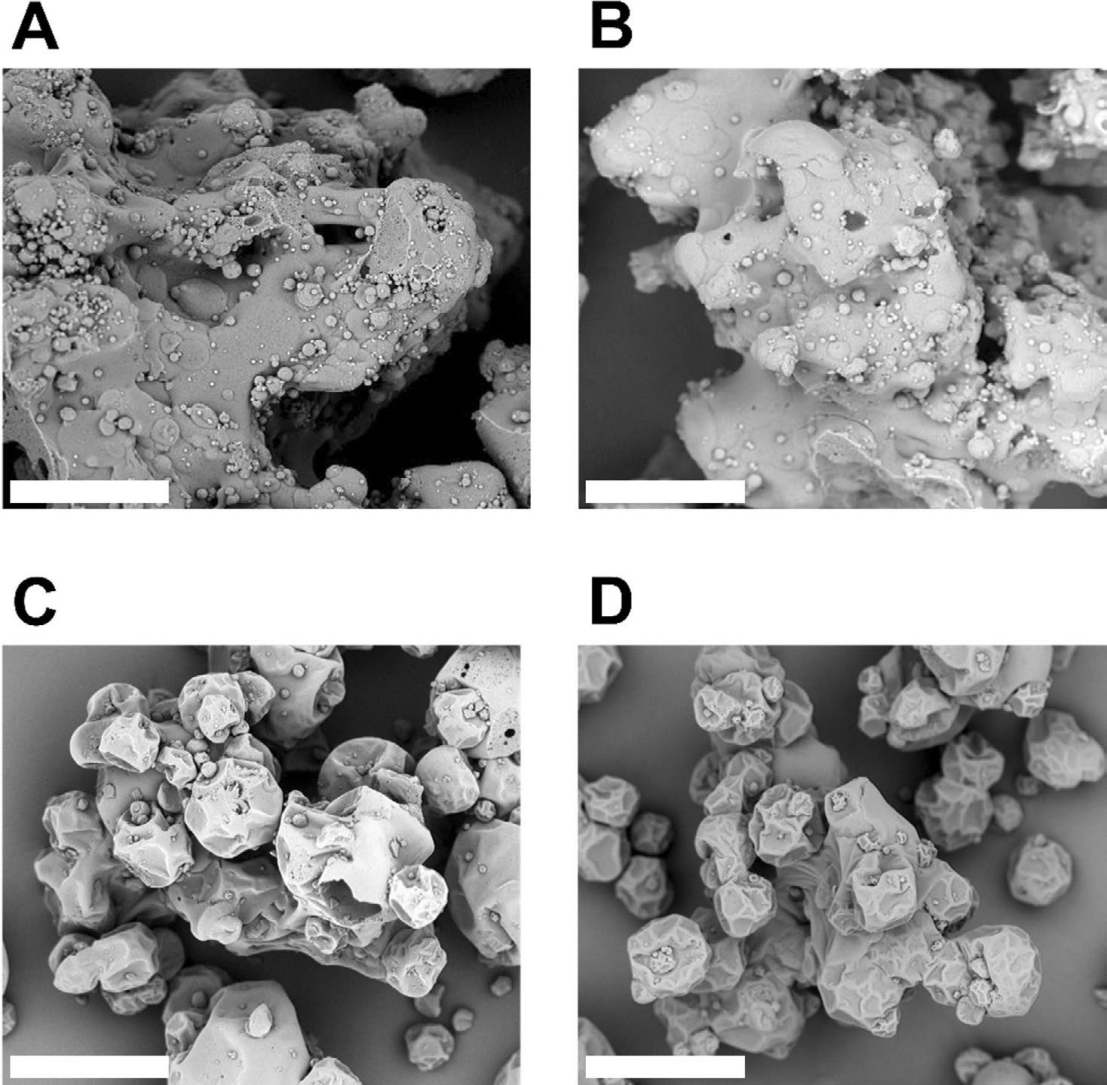
Morphologies of the different batches of PFH‐VAP microparticles. Scanning electron microscopy images recorded at 1000× magnification (scale bar = 80 μm) for the following batches: Panel A: PFH‐VAP250‐101, laboratory‐scale equipment; Panel B: PFH‐VAP250‐102, laboratory‐scale equipment; Panel C: PFH‐VAP250‐101, pilot‐scale equipment; and Panel D: PFH‐VAP250‐102, pilot‐scale equipment.

SEM images of the microparticles and particle size analyses (Figure 5 and Table 3, respectively) show that the particles dried using laboratory equipment were relatively large (D50 between 395 and 502 μm) and irregularly shaped owing to intended agglomeration of the atomized droplets and the starch during drying. The fine dots on the particle surfaces could therefore be either starch or spray‐dried droplets. In addition, drying on the pilot equipment produced relatively distinct spherical particles, which were formed during the initial spray drying stage and agglomerated owing to the recirculation of fines from the fluid bed (D50 between 86 and 102 μm).

## 4 Discussion

The system described herein represents the first use of BMC in food production. All ingredients present in the PFH‐VAP are GFSA‐compliant and can be obtained from multiple manufacturers/suppliers, thereby mitigating potential supply chain issues for commercial production.

Following optimization of the PFH‐VAP formulation and preparation at the laboratory scale and its successful transfer to the pilot manufacturing equipment, the different formulations were tested at the pilot scale. More specifically, the formulations were iterated and prioritized in terms of bouillon stability, with the two preferred formulations being identified based on their performances and the option of employing a naturally occurring antioxidant (TOC). Overall, the results demonstrate that the stability of PFH‐VAP in the bouillon cubes is superior to that of a commercial VAP product under harsh conditions (40°C/75% RH) both in the presence and absence of additional iron. The temperature and humidity conditions selected for evaluation were intended to comply with the ICH recommendations (International Conference on Harmonization 2003), while evaluating in an environment that is relevant to the populations that will consume PFH‐VAP‐fortified bouillons (i.e., low‐ and middle‐income countries with high bouillon consumption and elevated levels of VAD). However, it should be noted that the climatic conditions vary widely between and within countries; thus, future studies should involve the use of harsher conditions if considered relevant.

Notably, the developed process can be easily scaled up due to the fact that the emulsification and spray drying procedures are widely employed in food ingredient manufacturing. Importantly, the results demonstrate the consistent performance of PFH‐VAP after a 400× scale‐up from the laboratory scale to pilot‐plant scale. It is therefore anticipated that an additional scaling of 20–50× should be possible to achieve commercial production. Additionally, the use of the developed PFH‐VAP in bouillon is likely to have a positive cost impact, as the stability data suggest that less overage will be required to provide an appropriate amount of vitamin A. Overages are added at the time of fortification to compensate for anticipated stability losses prior to consumption.

Notably, one key obstacle to the use of VAP250 in bouillons is the lack of government regulations mandating the fortification of bouillons with vitamin A. Without mandates, food manufacturers or donors must subsidize the cost of fortification, or customers must be able to afford the cost increase. Other food vehicles, such as wheat flour and sugar, are currently mandated in multiple countries, thereby suggesting the possibility of applying the developed PFH‐VAP in these food items.

However, this development program had several limitations. For example, the stability studies evaluated the storage of samples under constant rather than two‐phase (day/night) environmental conditions. In addition, a single commercial VAP product was used for comparison, and only one bouillon recipe was examined. Comparisons with other commercial VAP products should therefore be considered in future studies.

The choice of bouillon as a proof‐of‐concept food vehicle was based on its wide consumption in the target population and its potential positive impact on micronutrient status and child mortality (Adams, Vosti, Becher, et al. 2024; Adams, Vosti, Somé, et al. 2024; Adams, Vosti, Tarini, et al. 2024; Thompson et al. 2024). Although the high salt content of bouillons (243 mg/g) can have negative health consequences, (Archer et al. 2022) recent research suggests that bouillons contribute to < 25% of the total salt consumed in the households of interest (Davis et al. 2024). Moreover, it should be emphasized that PFH‐VAP‐fortified bouillons may not provide the same benefit when used in acidic foods (such as tomato‐based foods) because BMC dissolves in acid and may lose its ability to protect VAP.

In addition, these formulations contain synthetic ingredients, including BHA and BHT. Although the antioxidants BHA and BHT are sometimes perceived negatively, it has been reported that the risk assessments of such ingredients may not always be relevant to human health, and that classifications sometimes misrepresent science (Felter et al. 2021).

BMC is also a synthetic material. This ingredient has been comprehensively studied and has been demonstrated to possess a low toxicity profile (Eisele et al. 2011). Current European Union (EU) regulations limit BMC consumption through food additives to 700 mg/d for adults (European Food Safety Authority Panel on Food Additives and Nutrient Sources Added to Food (ANS) 2010). Thus, considering that the recommended daily amount (RDA) of vitamin A is ~1.5 mg VAP/d, and the ratio of VAP to BMC is 1:1 in PFH‐VAP microparticles, fortification at levels reaching 100% of the RDA of vitamin A would result in BMC levels two orders of magnitude below the European food safety limit. Although the EU has implemented legislation (REACH Regulation (EC) No. 1907/2006) to restrict the use of synthetic polymer microparticles for environmental purposes, the regulations allow for the use of BMC in this food application as long as specific labeling and reporting requirements are followed. Furthermore, during its intended end use (i.e., consumption), BMC dissolves in the acidic conditions of the gastrointestinal tract. Therefore, it is expected that BMC will be excreted as single molecules rather than as intact microparticles. To quantify the relative risk–benefit of BMC's application in food fortification would require further environmental assessment.

Future efforts will focus on evaluating PFH‐VAP manufactured on a commercial scale and its use in fortifying bouillons. Particle size and flowability properties will be important to tune at commercial scale because food vehicles may have specific size or flowability requirements for fortificants. For example, flour has a smaller particle size requirement than sugar. Particle size and morphology requirements for bouillon may be different than either flour or sugar since bouillon is usually compressed into a tablet instead of remaining in a free‐flowing powder. Cost will be critically important to achieve widespread impact. Sensory studies are currently ongoing, and a clinical study involving healthy volunteers is planned to characterize vitamin A absorption from PFH‐VAP‐fortified bouillons.

## 5 Conclusion

PFH‐VAP, a spray‐dried BMC‐encapsulated form of VAP, was developed and optimized for its evaluation in stability studies. The stability of PFH‐VAP prepared at the laboratory scale and pilot scale outperformed that of a commercial VAP250 product when tested in bouillon cubes. The incorporation of PFH‐VAP could therefore improve the effectiveness of vitamin A food fortification programs by increasing vitamin A levels after storage and cooking.

## Ethics Statement

This study does not involve any human or animal testing.

## Conflicts of Interest

The authors declare the following potential conflicts of interest regarding the research, authorship, and/or publication of this article. Sam Brady, Don Chickering, and Haisong Yang are employees of PFH, a company developing PFH‐VAP. Elsa Abou Jaoude, Julie Wyns, and Jérôme Vallejo are employees of LIS contracted by the PFH to produce PFH‐VAP. Justyna Ebbesen and Elise Ivarsen are employees of Eurofins contracted by the PFH for sample analysis. Julie Straub is a consultant for PFH. Sam Brady, Don Chickering, Haisong Yang, Jérôme Vallejo, Julie Wyns, Elsa Abou Jaoude, and Julie Straub are inventors on PCT/US2023/065451 and US patent application #18/296,681.

## Supporting information


**Table S1:** Manufacturing and Analysis Equipment.
**Table S2:** Manufacturing Materials.

## Data Availability

The supporting data are openly available in Figshare: https://doi.org/10.6084/m9.figshare.28603928.v1. Data are available under the terms of the Creative Commons Attribution 4.0 International license (CC‐BY 4.0).
