# Supplementary material for: An Encapsulated Vitamin A Palmitate Powder With Improved Stability for Use in Food Fortification
Source: Food Sci Nutr. 2025 Nov 21;13(11):e71237. doi: 10.1002/fsn3.71237 (PMC12636930; doi:10.1002/fsn3.71237)
Supplement: Supplementary file 1 — Table S1: Manufacturing and Analysis Equipment. Table S2: Manufacturing Materials. [file FSN3-13-e71237-s001.docx]

**Supporting Information**

**An Encapsulated Vitamin A Palmitate Powder with Improved Stability for Use in Food Fortification**

Samantha Brady^1^, Elsa Abou Jaoude^2^, Julie Wyns^2^, Haisong Yang^1^, Justyna Ebbesen^3^, Elise Ivarsen^3^, Julie Straub^4^, Jérôme Vallejo^2^, Don Chickering^1^

1. Particles for Humanity, PBC, 44 Oak Ridge Rd, West Lebanon, New Hampshire, United States, 03784
2. LIS France, 67 Rue de la Gare, Cérences, France, 50510
3. Eurofins Steins Laboratorium A/S, Ladelundvej 85, Vejen, Denmark, 6600
4. Straub Pharma Consulting, Inc., 100 Cambridge St, Winchester, Massachusetts, United States, 01890

Corresponding author:
Samantha Brady
Particles for Humanity, PBC
44 Oak Ridge Road
West Lebanon, New Hampshire, 03784, United States
E-mail: sbrady@particlesfh.com

**Supplemental Table 1. Manufacturing and Analysis Equipment**

| **Description** | **Company** |
| --- | --- |
| APV Model 2000 high-pressure homogenizer | SPX Flow, Inc., Charlotte, North Carolina, USA |
| Ultra-Turrax T25 high-shear mixer | IKA Works, Inc., Wilmington, North Carolina, USA |
| Mini spray dryer B-290 | Büchi Corporation. New Castle, Delaware, USA |
| Spray dryer nozzle | Büchi Labortechnick AG, Flawil, Switzerland |
| Minilab RC fluidized bed dryer | Diosna Dierks & Söhne GmbH, Osnabrück, Germany |
| Fluid bed nozzle | Düsen-Schlick GmbH, Untersiemau/Coburg, Germany |
| Direct-Q 3 UV remote water purification system | Merck KGaA, Darmstadt, Germany |
| Pilot-scale tanks | ETA, Rians, France |
| MG2-350S high-pressure homogenizer | Bos Homogenisers B.V., Hilversum, The Netherlands |
| Rotina 420 centrifuge | Hettich North America, Beverly, Massachusetts, USA |
| IKA VIBRAX VXR basic | IKA–Werke GmbH & Co. KG, Staufen, Germany |
| IKA MS 3 basic | IKA–Werke GmbH & Co. KG, Staufen, Germany |
| UltiMate 3000 RP-HPLC | Thermo Fisher Scientific Inc., Waltham, Massachusetts, USA |
| Thermomixer C | Eppendorf, Hamburg, Germany |
| PowerPrep Plus, 14Qt | Cuisinart, Stamford, Connecticut, USA |
| Model C benchtop manual press | Carver, Inc., Wabash, Indiana, USA |
| 3 ton arbor press, Model 3Z949E | Dayton, Niles, Illinois |
| 15 mm cube punch and die (S7 ERS tool steel) | Natoli Engineering Co. Inc., St. Charles, Missouri, USA |
| 31 ×23 mm tablet punch and die (S7 ERS tool steel) | Natoli Engineering Co. Inc., St. Charles, Missouri, USA |
| Custom punch and die set for P40 rotary press | Elizabeth Europe, Blois, France |
| Kmix 750 stand mixer | Kenwood, Hachioji, Japan |
| P40 rotary press | Bonals Technologies, Saint–Just–Saint–Rambert, France |
| BCW3 wrapping machine | Theegarten-Pactec GmbH & Co. KG, Dresden, Germany |

**Supplemental Table 2. Manufacturing Materials**

| **Description** | **Supplier, location** |
| --- | --- |
| Laboratory-scale production of PFH-VAP | |
| L-ascorbic acid | Luwei Pharmaceutical Group through Prinova, London, UK |
| BMC | Evonik, Essen, Germany |
| VAP oil stabilized with BHT | DSM, Basel, Switzerland |
| VAP oil stabilized with tocopherol | BASF, Levallois–Perret, France |
| BHA | Merck, Sigma–Aldrich, St. Louis, Missouri, USA |
| Maltodextrin DE19 | Tereos, Aalst, Belgium |
| HI CAP 100 modified starch | Ingredion, Westchester, Illinois, USA |
| Native starch | Roquette, Lestrem, France |
| Pilot-scale production of PFH-VAP | |
| BHA | Merck, Sigma–Aldrich, St. Louis, Missouri, USA |
| BHT | Merck, Sigma– Aldrich, St. Louis, Missouri, USA |
| L-ascorbic acid | Luwei Pharmaceutical Group through Prinova, London, England |
| Reverse osmosis water | LIS France, Cérences, France |
| BMC | Evonik, Essen, Germany |
| Maltodextrin DE19 | Tereos, Aalst, Belgium or Roquette, Lestrem, France |
| CAPSUL TA modified starch | Ingredion, Westchester, Illinois, USA |
| VAP oil stabilized with TOC | BASF, Levallois–Perret, France |
| Unstabilized VAP oil | BASF, Levallois–Perret, France |
| PE-100 | Qingdao Nutrend Biotech Company Ltd,. Qingdao Shangdong, China |
| Bouillon studies | |
| Bouillon cubes (laboratory scale) | Nestle, Vevey, Switzerland |
| Maggi bouillon powder (pilot scale) | Nestle, Vevey, Switzerland |
| Iron pyrophosphate (pilot scale) | Spectrum Chemical Manufacturing Corp., New Brunswick, New Jersey, USA |
| Commercial VAP 250 Food Grade | BASF, Ludwigshafen, Germany |
| Wrappers | Huhtamaki Group, Espoo, Finland |

Abbreviations: BHA: butylated hydroxyanisole; BHT: butylated hydroxytoluene; BMC: basic methacrylate copolymer; TOC: tocopherol; VAP: vitamin A palmitate
